# Supplementary material for: MetaRibo-Seq measures translation in microbiomes
Source: Nat Commun. 2020 Jun 29;11:3268. doi: 10.1038/s41467-020-17081-z (PMC7324362; doi:10.1038/s41467-020-17081-z)
Supplement: Supplementary file 10 — Supplementary Data 7 [file 41467_2020_17081_MOESM10_ESM.zip › File2/Confidence_VeryHigh_Taxonomy/83315_out.krona.html]

Javascript must be enabled to view this page.

members
magnitude
magnitudeUnassigned
count
unassigned
taxon
rank

83315\_out

97

97
2
superkingdom

phylum
97
1239

class
186801
97

order
1
97
186802

SRS077231\_contig\_number\_contig-100\_38629.38630

2
186806
family

genus
1730
2

species
142586
2

SRS024075\_contig\_number\_16021SRS140492\_contig\_number\_17188


SRS063985\_contig\_number\_contig-100\_34154.34154
1
186803
94
family

genus
2
841


SRS016954\_contig\_number\_27855
1
360807
species

species

SRS044535\_contig\_number\_14583
2049040
1

10
572511
genus


SRS019968\_contig\_number\_contig-100\_744.98488SRS024435\_contig\_number\_6935SRS045004\_contig\_number\_33095SRS046369\_contig\_number\_contig-100\_208.87495SRS055966\_contig\_number\_contig-100\_958.959SRS056519\_contig\_number\_3476SRS063813\_contig\_number\_contig-100\_1558.1559SRS098061\_contig\_number\_contig-100\_458.38690SRS147977\_contig\_number\_13184SRS148874\_contig\_number\_14957
10
33039
species

species

SRS011239\_contig\_number\_23541SRS013098\_contig\_number\_31814SRS013951\_contig\_number\_38222SRS014412\_contig\_number\_8363SRS014855\_contig\_number\_contig-100\_374.101545SRS014948\_contig\_number\_23788SRS015190\_contig\_number\_14879SRS015486\_contig\_number\_contig-100\_8011.8012SRS015663\_contig\_number\_37057SRS015694\_contig\_number\_1940SRS015782\_contig\_number\_28909SRS016018\_contig\_number\_14196SRS016335\_contig\_number\_contig-100\_932.118045SRS017191\_contig\_number\_18570SRS017521\_contig\_number\_48770SRS018656\_contig\_number\_2456SRS018817\_contig\_number\_contig-100\_12125.87635SRS018984\_contig\_number\_27091SRS019068\_contig\_number\_83521SRS019161\_contig\_number\_25537SRS019267\_contig\_number\_6490SRS019381\_contig\_number\_contig-100\_2264.108863SRS019397\_contig\_number\_1198SRS020869\_contig\_number\_1416SRS022609\_contig\_number\_17387SRS022713\_contig\_number\_contig-100\_11057.32692SRS023715\_contig\_number\_27961SRS024492\_contig\_number\_13072SRS042628\_contig\_number\_30641SRS043001\_contig\_number\_3277SRS043411\_contig\_number\_13590SRS043667\_contig\_number\_contig-100\_1215.36744SRS043701\_contig\_number\_13165SRS043768\_contig\_number\_30616SRS047044\_contig\_number\_14888SRS048870\_contig\_number\_23118SRS049446\_contig\_number\_contig-100\_368.93406SRS049896\_contig\_number\_contig-100\_9031.141281SRS049959\_contig\_number\_26834SRS050422\_contig\_number\_contig-100\_374.202278SRS050925\_contig\_number\_contig-100\_263.193064SRS051031\_contig\_number\_contig-100\_934.159363SRS052697\_contig\_number\_17843SRS053398\_contig\_number\_1217SRS054905\_contig\_number\_14602SRS056259\_contig\_number\_3936SRS057478\_contig\_number\_8342SRS062654\_contig\_number\_28355SRS074586\_contig\_number\_contig-100\_3264.44037SRS077502\_contig\_number\_6110SRS097958\_contig\_number\_23246SRS098073\_contig\_number\_contig-100\_462.114284SRS098644\_contig\_number\_38969SRS098717\_contig\_number\_contig-100\_924.151077SRS103987\_contig\_number\_27049SRS1041036\_contig\_number\_contig-100\_3779.3780SRS1041144\_contig\_number\_4683SRS105153\_contig\_number\_contig-100\_1133.222917SRS1055022\_contig\_number\_17595SRS1055043\_contig\_number\_contig-100\_2805.2806SRS1055067\_contig\_number\_90SRS1055099\_contig\_number\_contig-100\_3226.3227SRS142503\_contig\_number\_46487SRS142599\_contig\_number\_2394SRS142890\_contig\_number\_45783SRS143342\_contig\_number\_18104SRS143372\_contig\_number\_12478SRS143598\_contig\_number\_21176SRS144135\_contig\_number\_35652SRS144506\_contig\_number\_47411SRS144537\_contig\_number\_35487SRS144714\_contig\_number\_21412SRS146764\_contig\_number\_34232SRS146812\_contig\_number\_contig-100\_292.321684SRS147377\_contig\_number\_699SRS148424\_contig\_number\_26570SRS149075\_contig\_number\_contig-100\_3557.77189SRS149244\_contig\_number\_4122SRS149325\_contig\_number\_contig-100\_218.114101SRS893252\_contig\_number\_contig-100\_10832.54944SRS971276\_contig\_number\_contig-100\_1901.34696
39491
81
